# Host hybridization enabled the emergence of a reassorted hantavirus lineage

**DOI:** 10.1371/journal.ppat.1014458

**Published:** 2026-07-28

**Authors:** Anton Labutin, Nadine Ritter, Guiscard Seebohm, Gerald Heckel

**Affiliations:** 1 Institute of Ecology and Evolution, University of Bern, Bern, Switzerland; 2 Institute for Genetics of Heart Diseases (IfGH), Department of Cardiovascular Medicine, University Hospital Münster, Münster, Germany; 3 Chembion, University of Münster, Münster, Germany; 4 Department of Drug Design and Pharmacology, University of Copenhagen, Copenhagen, Denmark; University of Kansas, UNITED STATES OF AMERICA

## Abstract

The exchange of genetic material between individuals is a key driver of evolution and diversification across most branches of life. Segmented viruses can exchange genetic material through reassortment of genomic segments. New viral strains that emerge from reassortments can have greater infection ranges and higher virulence, although concrete examples of the adaptive advantages of reassortants in nature apart from influenza remain rare. We studied here the evolutionary history and consequences of reassortment in Tula orthohantavirus (TULV) in a hybrid zone between evolutionary lineages of its reservoir host, the common vole (*Microtus arvalis*). Across 58 trapping sites and 127 infected voles, we detected 27 TULV reassortants in a 12.5 km broad zone at the contact of the parental TULV clades, resembling a viral hybrid zone concordant with the hosts’. Phylogenomic analyses revealed three independent reassortment events, but most of the host hybrid zone was dominated by a single strain with a reassorted M-Segment, which encodes the surface glycoprotein. We detected clade-specific variation in the glycoprotein’s N-terminal region consisting of five residues, two of which showed evidence of positive selection. *In silico* 3D modeling of seven glycoproteins confirmed that this N-terminal region has a unique and specific structure for each TULV clade and the dominant reassortants and is the only structurally variable region of the TULV glycoprotein. Our findings suggest that reassortment between the parental TULV clades in the contact region has resulted in a transgressive virus phenotype potentially adapted to hybrid hosts. This demonstrates the potential of zones of hybridization for the emergence of new virus strains with novel evolutionary trajectories.

## Author summary

Reassortment - the exchange of genome segments between viruses during co-infection - can accelerate viral evolutionary processes by producing genomes that combine genetic variation from both parental strains, although concrete examples of a reassortants’ adaptive advantages in nature apart from influenza remain rare. By studying the consequences of reassortment in Tula orthohantavirus in a hybrid zone between evolutionary lineages of its reservoir host, we demonstrate the first example of host-virus co-hybridization. Our results suggest that a single transgressive reassortant has displaced both parental viral clades throughout a 12.5 km strip at the center of the host hybridzone, highlighting the importance of hybrid zones as hotbeds for viral evolution and suggesting potential viral adaptation to hybrid hosts. We support this by characterizing the viral glycoprotein through *in silico* modeling and identifying a single understudied region at the start in the N-terminal ectodomain as a likely candidate for regulating host specificity in TULV strains. This region may have broader implications for other hantaviruses in predicting infection ranges and zoonotic potential, and is a prime target for further experimental characterization of the still enigmatic process of hantavirus infections.

## Introduction

A main advantage of viruses in overcoming the defense systems of their hosts consists in their extraordinary speed of evolution. The high mutation rates of RNA viruses provide sufficient genetic variability to evade many host defenses. Reassortment - the exchange of genome segments between viruses during co-infection - can further accelerate evolutionary processes by producing genomes that combine genetic variation from both parental strains. The reassortants can display drastically altered host specificity, fitness, and virulence [1]. While the adaptive effects and public health risks of reassortments are best-documented for influenza viruses [2–5], reassortants have been observed across all segmented viral families [1,6,7].

Detecting the frequency of viral reassortments in nature and resolving their evolutionary histories and adaptive potentials poses significant challenges. Many reassortments probably remain undetected, because sequence divergence between the contributing strains is low and the fitness consequences for reassortants may be negligible. Additionally, reassortants that are less fit due to incompatibilities between segments [1,8,9] are likely purged over time and may disappear unnoticed. Reassortants that do persist are likely to be either selectively neutral or confer fitness advantages within their specific genetic backgrounds [1,10]. The potential fitness advantage gained through reassortment can be immediate rather than gradual via mutation. These advantages can be best seen in strains that persist through disruptive changes in the viruses’ environments; e.g., through spill-over into a new host or proliferation in genetically diverse hybrid hosts [11,12].

In this study, we examined the evolutionary history and the consequences of reassortment in hantaviruses in a zone of natural admixture through hybridization between genetic lineages of the reservoir host (hybrid zone). Hantaviruse*s* are trisegmented, negative-stranded RNA viruses that are most commonly associated with small mammals, particularly rodents (see [13] for a complete list of currently known hantavirus-host pairs). Some hantavirus species have repeatedly caused zoonotic outbreaks with high infection fatality rates, whereas others cause only mild – if any – symptoms [14]. Reassortments in hantaviruses appear to be rare in nature, with only a few cases described based on phylogenetic analyses [15,16], but comprehensive analyses are hindered by the limited availability of full genome data. Currently available observations indicate that hantaviruses most commonly reassort in nature with closely related strains, rarely between deeply divergent phylogenetic clades, and almost never with species from different hosts, even in the case of spillover infections [15]. Under laboratory conditions, it has been shown that reassortment in hantaviruses typically involved the medium (M)-segment encoding the surface glycoprotein [17]. Reassortment involving the large (L)-segment encoding the RNA-dependent polymerase and small (S)-segment encoding the hull protein might have more severe consequences for essential replication processes [17]. Reassortants in both nature and under laboratory conditions, however, generally lack any characterization in regards to their fitness or cellular interactions when compared to their parental hantaviruses.

Here, we explored the potential of genetic admixture in natural host hybrid zones to generate functionally relevant variation for hantavirus evolution. In Tula orthohantavirus (*Orthohantavirus tulaense*, TULV), the phylogenetic clades TULV-CEN.S and TULV-EST.S are functionally restricted to the respective Central and Eastern host lineages in European *Microtus arvalis* rodents, the reservoir host species [18,19]. Despite continued hybridization between *M. arvalis* lineages and extensive movement across the zone, the spatial transition between TULV clades is at most a few kilometers wide in the open landscape, suggesting very strong fitness impediment in the foreign host lineage [18–20]. No evidence of reassortment has been detected in the hybrid zone despite the presence of hosts infected with both virus clades (but see [21]). This suggests very high fitness costs and/or a low frequency of TULV reassortment in nature even among sister clades with a level of divergence that is well below the criterion of the International Committee on Taxonomy of Viruses (ICTV) for distinct virus species.

We tested this hypothesis with detailed analyses of a new evolutionary replicate of TULV sister clades across the hybrid zone between the Central and Eastern lineages of *M. arvalis* rodents. By utilizing full genome information and protein modeling of TULV, we trace the evolutionary history and potential adaptive consequences of reassortments between these two virus clades. Our analyses show that reassortment can lead to a “transgressive” hantavirus phenotype - a term borrowed from plant and animal hybridization, referring to a novel hybrid phenotype with advantageous characteristics not observed in parental lines - that confers advantages particularly in hybrid hosts.

## Results

**Fig 1:**
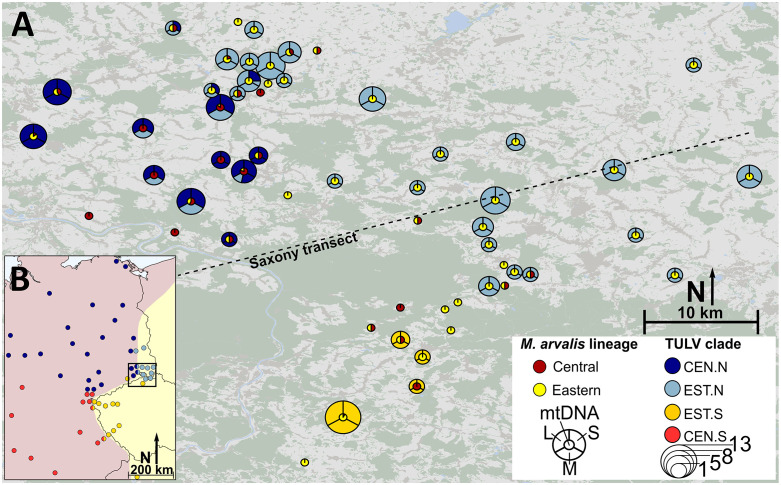
Zone of hybridization between Tula orthohantavirus (TULV) clades associated with hybridizing rodent host lineages. **(A)** Sampling sites of common voles (*Microtus arvalis*) across the Saxony transect. Circle sizes correspond to the numbers of TULV infected individuals. Small symbols lacking the outer circle represent sampling sites without infected individuals. The background map shows bodies of water in blue, settlements in brown, forests in green and open area in grey. The dashed line indicates the axis of the Saxony transect. Topographic backgrounds were modified after the osmdata package [24] and the original mapfile can be found under https://osmlanduse.org/#12/8.7/49.4/0/ and their licensing here: https://www.openstreetmap.org/copyright. **(B)** Geographical context of our study area (square) in eastern Germany in the hybrid zone of the Central and Eastern evolutionary lineages of the common vole. The displayed countries are Germany (left), Poland (top right), the Czech Republic (central right) and Austria (bottom right). The distribution of the clades TULV-CEN.N (blue), TULV-CEN.S (red), TULV-EST.S (yellow) and TULV-EST.N (light blue) is shown across Central Europe based on [18,20,21,25].

Sampling of 1284 common voles within the hybrid zone between the Central and Eastern evolutionary lineages revealed two narrow geographic contacts between three major phylogeographic clades of TULV (Fig 1). Consistent with earlier work further south in the hybrid zone [18,19,22,23], genetic screening of 183 common voles from 58 sampling locations confirmed a gradual transition in the frequency of mitochondrial cytochrome b (mtDNA) from the Central lineage (n = 51) towards the Eastern host lineage (n = 132) (Figs 1, S1, S1 Table).

Molecular screening of 528 adult voles identified 127 TULV positive individuals at 40 locations. Phylogenetic clustering based on partial S-segment sequences (see Materials and methods) assigned virus strains to the clades TULV Central North (TULV-CEN.N; n = 49), TULV Eastern North (TULV-EST.N; n = 61) and TULV Eastern South (TULV-EST.S; n = 17) (Figs 1, S2 and S1, S2 Tables). The northern part of the study region contained a geographically narrow contact between the clades TULV-CEN.N in the west and TULV-EST.N in the east (Fig 1). The presence of TULV-EST.S in the southern part of the study region is consistent with earlier data [18–20]. Vast forests limit the dispersal of common voles and probably TULV towards the north [18,22,23,26].

Further sequencing of partial M- and L-segments (see Materials and methods) demonstrated consistent assignments to the clade TULV-EST.S for all 17 samples, and to TULV-CEN.N and TULV-EST.N, respectively, for 84 infected voles (Fig 1). However, we detected 26 cases of mismatch in clade assignments between partial genome segments, representing potentially reassorted virus genomes (see below). The respective vole hosts originated from eight locations in a 12.5 km broad region at the immediate contact between the TULV-CEN.N and TULV-EST.N clades (Figs 1, S2).

**Fig 2:**
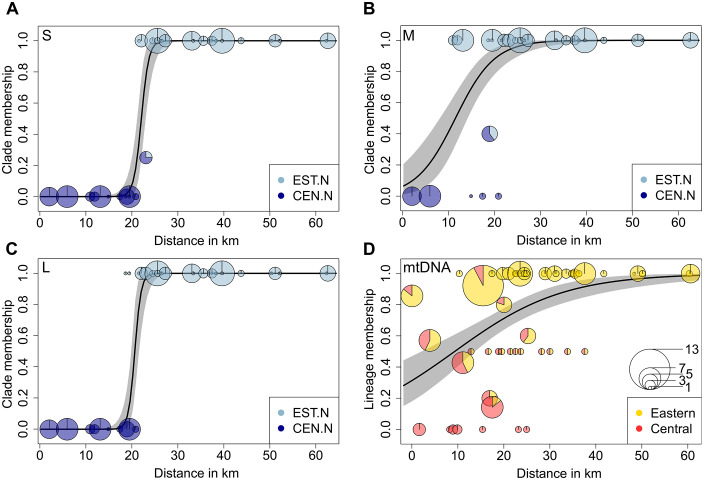
Geographic clines through the hybrid zone of the TULV-CEN.N and TULV-EST.N clades and the evolutionary lineages of the rodent host. The y-axis shows the average membership towards the TULV clades **(A-C)** or host lineages **(D)** for each sampling site. With the exception of the clines of the S- and L-segment (A & C), all clines showed significant differences in widths and centers (S2 Table). 95% confidence intervals are shown in grey. Distances are relative to the western end of the transect. Circle sizes correspond to the number of samples per site.

Geographic cline analysis revealed significant differences between the spatial distributions of virus genome segments across the host hybrid zone (Fig 2, Table 1). While the S- and L-segments displayed concordant narrow cline widths (3.3 km and 2.7 km, respectively), the cline for the M-segment was significantly broader (17 km) and shifted 10 km eastward (Fig 2, Table 1). The cline for host mtDNA between Central and Eastern lineages was even broader (47.4 km) and differed significantly from all TULV geographic clines (Table 1), which is consistent with the patterns detected in study transects further south (see [18]).

**Table 1:** Geographic cline analysis summary. The position of cline centers and their widths are given in km from the western end of the Saxony transect. Only estimates for the best supported model for each TULV genome segment and host mtDNA are shown. 95% confidence intervals are presented in brackets.

	S-Segment	M-Segment	L-Segment	mtDNA
**Center**	22.05 (21.08 - 23.05)	11.47 (7.63 - 14.36)	20.63 (19.83 - 21.59)	9.25 (1.13 - 13.92)
**Width**	3.25 (1.78 - 5.98)	16.99 (11.31 - 27.43)	2.74 (1.51 - 5.42)	47.35 (32.46 - 77.93)

**Fig 3:**
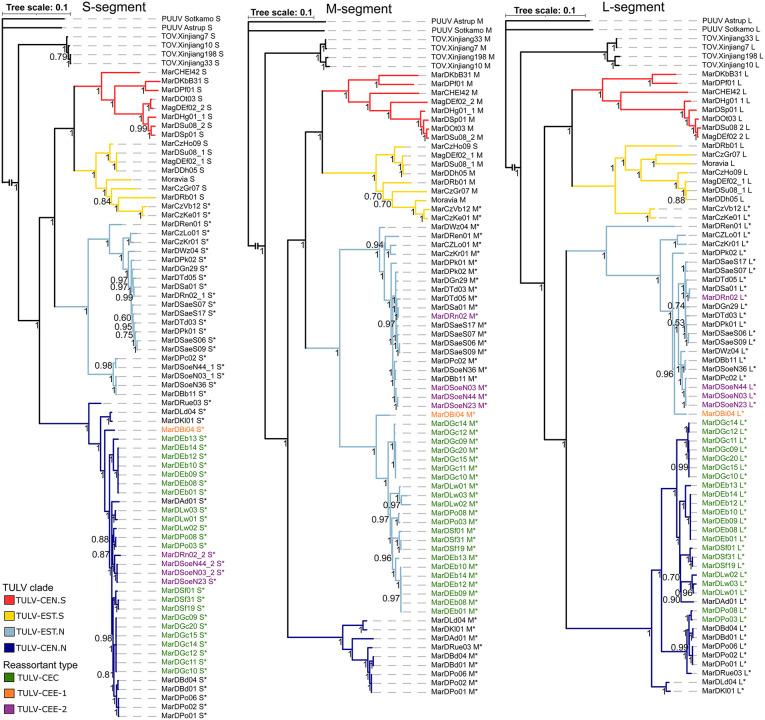
Phylogenetic relationships of complete TULV genome segments. The analysis was based on the complete coding region of each TULV segment with Puumala orthohantavirus (PUUV) as the outgroup. Names with an asterisk show new TULV genome sequences from this study. Sample names of reassorted genomes are highlighted by color according to the type of reassortment. Bayesian posterior probabilities are included for all nodes. The scale bar on top shows evolutionary distance in substitutions per nucleotide. For better visualization, branches towards the outgroup were truncated, indicated by double slashes through the branch. Evolutionary distance between PUUV and TULV is approximately 10 times higher than shown.

Focusing on the area of potential reassortment, whole genome sequencing (WGS) of 55 TULV samples (see Materials and methods, mean read depth 795x; S5 Table) confirmed concordance of clade assignment patterns for full-length genome segments with partial S-, M-, and L-segment sequences (Figs 3, S2). A total of 27 reassorted TULV were identified, as WGS also revealed an additional reassorted M-Segment that could not be amplified for partial sequencing. The most common reassortment pattern was detected in 22 voles at five locations and involved S- and L-segments from TULV-CEN.N combined with an M-segment from TULV-EST.N, thus the designation CEC. Five additional reassortants designated CEE contained S-segments from TULV-CEN.N and M- and L-segments from TULV-EST.N.

The high resolution of full genome sequences enabled us to phylogenetically identify at least three reassortment events between the TULV sister clades. The reassortant type CEC contained M-segments that were distinct from all other parental clade M-segments, while the involved S- and L-segments were similar to other parental clade strains from the region (Fig 3). The five TULV with reassortment pattern CEE represented two distinct reassortment histories as demonstrated by the phylogenies (Fig 3). CEE-1 contained an M-segment most similar to the ones in CEC, but an L-segment from non-reassorted TULV-EST.N. This suggests that it originated from a secondary reassortment event between parental TULV and a CEC strain (Fig 3). The third reassortment pattern CEE-2 was carried by four common voles: One sample contained a complete S-segment from TULV-CEN.N with M- and L-segments from TULV-EST.N, while the other three samples contained complete S-segments from both virus clades. Phylogenetic reconstructions based on amino acid sequences showed patterns of clustering analogous to those in nucleotide sequences (S3 Fig).

Phylogenetic dating with BEAST allowed us to estimate the time to the most recent common ancestor (TMRCA) of the defining reassorted M-segment from the TULV-CEC and CEE-1 strains and parental TULV-EST.N at ~104.6 years (S4 Table). For comparison, the TMRCA of the two reassortants was estimated at ~ 33.7 years. The former estimate aligns roughly with those reported for clade splits between other TULV clades, which likely strongly underestimate true evolutionary timescales due to mutational saturation [27]. The historical split between parental and reassorted M-segments of TULV-EST.N was supported by a sliding window analysis of complete coding sequences (cds), showing elevated levels of nucleotide diversity in line with those between different clades (Table 2, Fig 3).

**Table 2:** Divergence between clusters of the TULV-EST.N M-Segment. The table shows nucleotide diversity within the two different clusters of the complete M-Segments in the Saxony transect, TULV-CEC & TULV-CEE-1 (n = 23) and TULV-CEE-2 & parental TULV-EST.N (n = 21) and net nucleotide divergence between them. Results are shown for the coding nucleotide sequence (nt), the amino acid sequence (AA), and d_N_/d_S_.

		M-Segment
	**Length**	3423
**TULV-CEC & TULV-CEE-1**	**nt**	2.43%
	**AA**	0.32%
	**dN/dS**	0.013
**TULV-CEE-2 & TULV-EST.N**	**nt**	1.79%
	**AA**	0.20%
	**dN/dS**	0.011
**Between clusters**	**Nuc**	13.04%
	**nt**	0.97%
	**dN/dS**	0.01

**Fig 4:**
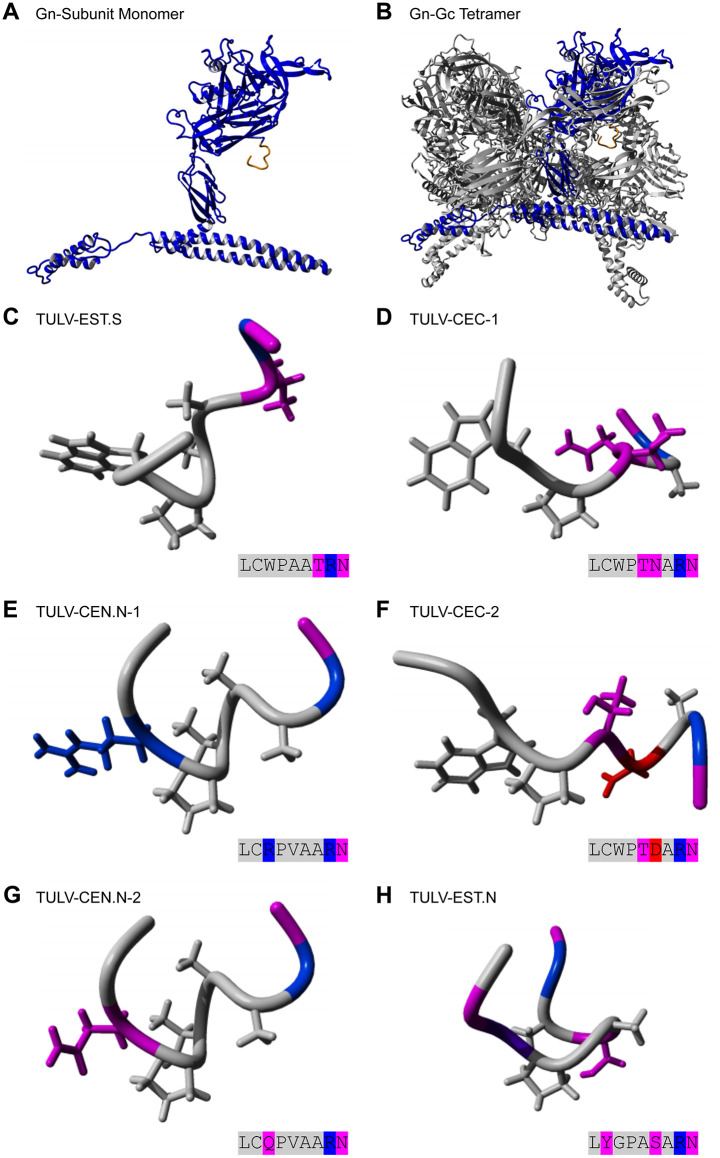
TULV surface glycoprotein structure and variation in its N-terminal ectodomain. **A)** Secondary structure of the Gn-subunit of the TULV surface glycoprotein from the reference genome Moravia (TULV-EST.S), with the ectodomain at the top and the transmembrane domain at the bottom. **B)** Gn-Gc tetramer structure of the mature TULV glycoprotein. The first Gn-subunit is shown in blue in an analogous orientation to A) with the remaining protein shown in grey. The region displayed in **C - H)** is colored in orange in the first Gn-subunit of A) and B). C - H) Close-ups of different variants of the N-terminal ectodomain of the Gn-subunit. C) Represents the identical structure for both Moravia and the TULV-EST.S genome from the Saxony transect. All structures were aligned along the conserved Proline residue 16 for identical orientation. C - H are colored based on the CPK convention for amino acids: hydrophobic - grey, polar - magenta, acidic - red, basic - blue.

Extremely low dN/dS ratios across most of the TULV genome indicated strong purifying selection as the main evolutionary force, with a single exception: a highly variable five-amino-acid region (residues 15–19) at the N-terminal ectodomain of the M-segment (S4, S5 Figs see also [18,20]). Positive selection was detected at residue 18 (Fast, Unconstrained Bayesian AppRoximation (FUBAR) and Mixed Effects Model of Evolution (MEME)) and residue 15 (FUBAR only) (S5, S6 Tables). These sites encode amino acids covering the entire spectrum of variable biochemical properties (Fig 4), indicating that this region is likely to have a different profile of molecular interactivity across the TULV clades and reassortants.

Protein modelling of six unique M-segments from the sampling region, as well as the TULV reference Moravia [28] showed an overall highly conserved structure of the glycoprotein, with the sole exception of the N-terminal ectodomain (Fig 4 and S10 Table). Pairwise root mean square deviation (RMSD) values for this region were strongly elevated compared to the rest of the protein, indicating substantial structural divergence. All models consistently predicted a signal peptide spanning residues 1–12 followed by a mature glycoprotein starting at residue 13 and terminating at residue 1104. Across the glycoprotein, variability was mostly confined to the residues 13–22, except for TULV-EST.S, which exhibited additional variation at residues 402–411. In the folded TULV-EST.S glycoprotein, this second region was located directly opposite the N-terminal ectodomain and was likely influenced by its structure. Root mean square fluctuation (RMSF) analysis of the ectodomain identified residues 15, 17, and 18 as the most flexible sites, suggesting high interaction potential (S6 Fig). No correlation was found between charge or polarity of residues and RMSF, implying structural rather than electrostatic differences in the different ectodomains.

## Discussion

Our study presents a novel case of host-virus co-hybridization, previously only observed in eukaryotic parasites and their hosts [29]. Within the hybrid zone, the reassortant TULV-CEC has largely displaced both parental viral clades. The dominant reassortant contains a surface glycoprotein which exhibits distinct structural variation and positive selection signals in its N-terminal ectodomain. Our findings suggest that this reassortant represents a transgressive phenotype potentially specifically adapted to the hybrid host environment, highlighting hybrid zones as dynamic settings for viral evolution and adaptation.

### Adaptive reassortment in TULV

The persistence of reassortants in natural populations requires continued genomic compatibility based on highly specific RNA-RNA and RNA-protein interactions [1]. Most reassortants between deeply diverged viruses cannot maintain this compatibility, thus suffer fitness costs and are quickly purged from populations [1,9]. Similarly, hybridization in hosts can impose additional fitness constraints on associated parasites due to novel genetic and immunological environments [11,12]. Despite these hurdles, we observed a reassorted hantavirus (TULV-CEC) that not only persisted but has become dominant within a 12.5 km broad region of the host hybrid zone, displacing both parental clades. This pattern suggests that the TULV-CEC M-segment provides a fitness advantage in hybrid hosts. Concrete verification of this hypothesis would, however, require further testing in vitro. Viral replication and shedding of different TULV clades and the reassortant would need to be quantified in cell cultures or living hosts of different genetic backgrounds to confirm a fitness advantage of the reassortant in hybrid hosts (see [30]).

The deep phylogenetic divergence between the M-segment of the CEC reassortant and other TULV-EST.N M-segments from the region (Fig 3) suggests evolution within hybrid hosts for an extended period of time. Viral reassortments with adaptive functions have been primarily observed for the influenza A virus [4,31,32], but are also sporadically documented for a variety of other viral families, including *reoviridae* [33] and *peribunyaviridae* [34]. Among the hantaviruses, reassortments documented in nature so far have been attributed to selectively neutral processes [15]. However, the few studies that examined hantavirus reassortment in nature lack the in-depth characterization necessary to establish their adaptive potentials [15,16,35,36]. Our findings suggest that reassortment-driven adaptation in hantaviruses may be more common than previously recognized.

### Additional reassortment types

The presence of multiple reassortment types within the TULV-CEN.N/TULV-EST.N contact zone suggests a higher degree of segment compatibility than for previously reported TULV clade interactions [18–20]. While M-segment reassortment is the most frequently observed form in hantaviruses [15], we also identified reassortants involving the S- and L-segments (TULV-CEE-1 & 2, Fig 3). TULV-CEE-2 consists of segments, which are nearly identical to local variants in the same population, suggesting that the causative reassortment event occurred not long ago (S4 Table). Notably, three out of four samples contained S-segments from both parental virus clades (Fig 3). These three might represent hosts with double infections for TULV-EST.N and TULV-CEE-2, in which case the M- and L-segment between both could be too similar to distinguish. Alternatively, these three may represent transiently diploid genomes for the S-segment. Transient segment diploidy has been observed *in vitro* as an intermediate state during reassortment [17,37,38]. These viruses are expected to eventually segregate into stable reassortants or parental genotypes [38], which may have already occurred in the haploid TULV genomes from the TULV-CEE-2 sampling sites.

### Structural variation and functional implications

Our results suggest that structural differences in a short region at the N-terminus of the TULV glycoprotein may be responsible for an adaptive advantage of TULV-CEC, resulting in its spread in the hybrid zone. This genome region exhibits substantial structural and genetic divergence (Figs 4, S4 & S5). We observed strong signals of positive selection at key residues (S8 & S9 Tables) that are in line with previous reports [18,20], all of which point to this region being a hotbed of evolutionary activity. In TULV, this region is part of the N-terminal ectodomain of the Gn-subunit [39,40]. Protein modeling revealed that the N-terminal ectodomain forms a distinct and consistent shape for each TULV clade, positioned within an exposed surface pocket between two glycoprotein spikes (Fig 4B). The functional role of this region remains unclear, but its spatial localization suggests a role in host receptor binding or immune evasion. The N-terminal domains of the Gn-Gc subunits have been implicated in glycoprotein maturation, viral entry, and immune interactions in other hantaviruses [41–44]. Experimental verification of this regions function could entail initial deletion followed by an analysis of post-deletion fitness to verify a concrete role in hantaviral infection. This could be followed up by an assay on potential interaction partners to confirms its role in the metabolic pathways of viral infection, replication and transmission.

Our models predicted the start of the protein at residue 13, preceded by a signal peptide of 12 residues. However, predictions of the protein start are inconsistent across hantaviruses and individual studies. The TULV M-segment coding region starts 15 bp upstream compared to other hantaviruses, aligning our predictions with the peptide start at residue 18 first described via protein crystallography of the Haantan virus [45]. Crystallography studies initially placed the start at residue 25 in Puumula virus (PUUV) [46], but later also revised it to 18 [47]. Other orthohantaviruses show further variation: Maporal virus (residue 22) and Andes virus (residue 23) [48]. These inconsistencies have led to a general neglect of the N-terminus in hantavirus glycoprotein studies. However, the absence of structural variation in other parts of the protein suggests that this region could play a primary role in shaping the adaptive landscape of TULV reassortants and may carry a similar level of importance across the entire hantavirus family.

### Conclusion

Our findings provide strong evidence for adaptive reassortment in a non-pathogenic hantavirus, driven by hybridization of its rodent host, and highlights the importance of fine-scale ecological and genetic context in understanding viral adaptation. The emergence and persistence of the reassorted TULV-CEC strain suggests that under the right conditions, reassortment can act as a mechanism for viral adaptation to novel host environments in hantaviruses. Hybrid zones, as natural settings of elevated and recombined genetic diversity of hosts, may play an underappreciated role in facilitating viral evolution and should be considered as key sites for studying the emergence of new viral phenotypes. Furthermore, our structural analyses revealed that a short region at the start of the N-terminal ectodomain of the TULV glycoprotein is a hotspot of adaptive change, suggesting a key role in host-virus interactions and emphasizing the need for further functional characterization of this region.

## Materials and methods

### Sample acquisition

A total of 1284 common voles *(Microtus arvalis*) were collected from 58 trapping locations at the border of Germany and the Czech Republic (Fig 1). This region, termed the “Saxony transect”, was expected to encompass the hybrid zone between the Central and Eastern evolutionary lineages of the vole host, as well as the contact zone of the TULV clades TULV-CEN.N, TULV-EST.N, and TULV-EST.S, as suggested by prior studies [18,19,25]. Snap traps were used for vole collection, and specimens were stored at -20°C immediately after retrieval. All samples are archived at the Institute of Ecology and Evolution at the University of Bern.

### TULV screening and phylogenetic analysis

528 adult common voles from 56 sites were screened for TULV infections. Only adult voles weighing at least 20g (summer) or 18g (autumn) were included, as juveniles show low TULV prevalence (<1%), likely due to maternal antibodies and limited exposure [49]. RNA was extracted from lung tissue using a modified QIAzol protocol [25]. TULV infection status was determined by RT-PCR amplification and Sanger sequencing of at least 427 base pairs (bp) of the S-segment nucleocapsid gene, following the assay described in [50]. A single sample (MarDSl04) could be amplified via RT-PCR, but not at sufficient quantity or quality for successful Sanger sequencing (S1 Table).

For reassortment detection, all TULV-positive samples underwent additional PCR amplification of M- and L-segments using primers C1/C2 [51] and HanLF1/HanLR2 [52], respectively, yielding fragments of at least 356 bp (M-segment) and 305 bp (L-segment). In the case of the M-segment, three individuals yielded no results using Sanger sequencing, although subsequent whole genome sequencing of one of the samples showed a complete TULV genome including the M-segment. To prevent loss of information for phylogenetic clade assignment due to the complete deletion of sites containing one or more missing or ambiguous nucleotides, a total 0.64% of sequence content was imputed based on the closest genetic relative [20]. Phylogenetic assignment of these segments was performed with MrBayes v3.2.7a [53] on the CIPRES platform [54]. We performed MCMC sampling for up to 10^8^ generations in four independent runs comprising four chains, implementing reversible-jump sampling over the entire general time-reversible substitution model space [55]. After discarding a burn-in fraction of 25%, samples were recorded every 10^3^ generations. Chains converged after 2 460 000, 1 965 000 and 5 410 000 generations for the S-, M- and L-segment respectively. Phylogenetic trees were drawn and edited using the online platform iTOL v5 [56]. We created maps for visualization of the viral clade distribution using the geosphere package [57] in R and topographic backgrounds were modified after the osmdata package [24].

### Sequencing of host mitochondrial DNA

We extracted host DNA according to a standard phenol-chlorophorm protocol (modified after [58]). We used mtDNA in order to assess the evolutionary lineages of voles across the Saxony transect. Host evolutionary lineage was determined by sequencing [59] at least 458 bp of the mitochondrial cytochrome b gene in 183 individuals. All TULV infected individuals, as well as a minimum of two individuals per population when available were sequenced to show the distribution of host lineages in the Saxony transect. Phylogenetic analysis followed the same pipeline as viral segments, with lineage assignment based on reference sequences [26]. Chains converged after 12 850 000 generations. The information on mtDNA lineages was used in context with previous publications on the Central-Eastern *M. arvalis* hybrid zone as a proxy for establishing genetic admixture and hybridization of the host in the Saxony transect [18–20,22]. Mitochondrial, sex-chromosomal and autosomal markers are geographically consistent in assigning evolutionary lineages of *M. arvalis* across Europe [26,59–63] except in the immediate zone of hybridization. The extensive mixture between mtDNA lineages within populations across the sampling region cannot be explained without a history of hybridization between evolutionary lineages, given the low mobility and dispersal distance of common voles [64,65].

### Geographic cline analysis

To assess the spatial transition of vole evolutionary lineages and TULV clades, geographic cline analyses were performed using the HZAR package in R [66]. Sampling locations were projected onto a one-dimensional transect axis, optimized to minimize geographic distance between the TULV-CEN.N and TULV-EST.N clades (Fig 1) [18,20,22]. Genomic segments from TULV-EST.S were not included in the analysis. Distances are given between the projection points and the western transect start in km. The proportion of each TULV clade (based on S, M, and L-segments) and host mtDNA lineage at each site was modeled using four cline models with increasing complexity: null (no cline), model 1 (free cline center and width), model 2 (free minimum and maximum frequency), and model 3 (additional exponential tail parameters). Likelihood scores of all cline models were compared for each analysis and cline parameters were estimated for the model with the highest likelihood, performing 10^5^ generations of MCMC sampling in three independent chains and with a burn-in period of 10^4^ iterations. We tested pairwise concordance of cline centers and widths across all analyses using a likelihood-ratio test (LRT) in R. We compared a null model of a concordant cline through two combined datasets to an alternative model of individual cline widths and centers for both datasets. We used two times the difference between the log-likelihoods of the alternative and the null models as the test statistic. Significance was determined based on a χ2 distribution with two degrees of freedom.

### TULV whole genome sequencing

To confirm reassortment patterns, whole-genome sequencing was performed on all individuals with partial segment evidence of reassortment, as well as on additional individuals from populations within 10 km of the clade contact and four reference sequences. Preparation of libraries, whole genome sequencing and genome assembly followed the hybrid sequence capture protocol in [67], using custom baits to capture and enrich viral sequences in libraries. Libraries were sequenced on an Illumina MiSeq (Illumina, San Diego, CA, USA) with 2 x 300 cycles by the Next Generation Sequencing Platform of the University of Bern. Hybrid sequence capture generated a total of 2,695,193 TULV sequence reads (596–192,233 per sample, median = 35,919), with an average read depth of 795× (range: 7×–2494×) across genomes (S5 Table). De novo genome assembly was conducted using Iterative Virus Assembler [68] and subsequently mapped back against the initial viral consensus genomes in order to infer quality and mapping statistics. Missing nucleotides in three genomes (MarDLw01, MarDLw02, MarDLw03) were imputed based on their closest genetic relatives. We calculated the percentage of sites with a read depth of at least 3x and 20x, average genomic coverage and total read count for each genome using R.

### TULV phylogenetic analysis

We carried out a phylogenetic analysis of TULV genomes both for the complete cds and the derived AA sequences of each viral segment from all genomes. Reference sequences consisted of published TULV genomes with a complete cds from Central Europe [18,28,67]. Four TULV genomes from *M. obscurus* in China and two PUUV were additionally included as outgroup genomes [69–72]. Bayesian phylogenetic inference for nucleotide sequences was performed as described for segment analysis. Chains converged after 670 000, 965 000 and 360 000 generations for the S-, M- and L-segment respectively.

For AA sequences we conducted the phylogenetic analysis in MEGA X [73]. We derived tree topologies using the Maximum Likelihood method based on the JTT matrix-based model [74] with 1000 bootstraps. Initial trees for all segments were obtained by applying Neighbor-Joining and BioNJ algorithms to a matrix of pairwise distances estimated using the JTT model and keeping the topology with the superior log likelihood value. Tree drawing and editing was performed identically to the TULV segment fragments (see above).

### TULV sequence diversity and signatures of selection

Genome wide nucleotide diversity and divergence between TULV clades was calculated across the cds of all TULV segments in DnaSP version 5 [75]. Sliding-window analyses (30-bp window, 10-bp step) were used to evaluate d_N_/d_S_ ratios (ratio of non-synonymous to synonymous substitutions) and D_XY_ (average number of nucleotide substitutions per site). AA divergence in the form of mean p-distance within and between TULV clades was calculated in MEGA X. Signatures of selection were inferred using the branch-site model [76] of CodeML, which is part of the PAML package version 4.9 [77]. The RAxML software version 8.2.12 on the CIPRES platform was used for the construction of phylogenetic trees for selection inference. Model likelihoods were compared to a null hypothesis using a likelihood ratio test and χ2 distribution. We used Bayes Empirical Bayes (BEB) [78] inference to identify sites under positive selection. We performed two additional scans for selection to test for rate variation at synonymous sites using FUBAR [79] and MEME [80] in HYPHY [81] on the Datamonkey webserver [82]. Posterior probabilities > 0.85 or p < 0.1 were considered as evidence of positive selection for sites.

### Phylogenetic dating of TULV

Estimates for the time of most recent common ancestor (tmrca) of clade and cluster splits within TULV were calculated using the BEAST v1.10.4 software [83]. We followed the parameters established in [27], using a substitution rate of 1.51 × 10^−3^ substitutions per site per year for TULV, as well as a slower substitution rate estimate for hantaviruses of 2.7 × 10^−4^ [70]. The latter substitution rate naturally led to estimations of a notably higher TMRCAs, but proportions of TMRCAs between clades and clusters were consistent between higher and lower substitution rates. Only estimates for 1.51 × 10^−3^ are shown in the results, as they are directly comparable to [27]. We implemented a Bayesian skyline coalescent tree prior alongside a relaxed lognormal molecular clock for MCMC sampling of 10^8^ generations. Samples were recorded every 5000 generations.

### Homology modeling, structural alignments and molecular dynamics simulations

To assess structural differences in the M-segment glycoprotein among TULV clades and reassortants, we conducted homology modelling and 3D visualization of the TULV M-segment and its N-terminal ectodomain. We selected six M segment sequences which reflect the full diversity spectrum for AA in the N-terminal ectodomain and over 90% of all AA diversity across the entire M-Segment from the Saxony transect, as well as the original TULV genome Moravia [28] as a TULV-EST.S reference. Homology modelling and molecular dynamics (MD) simulations were performed in YASARA Structure version 20 using adapted macros and structures implemented from [84,85]. Homology models were built using the standard “hm_build.mcr” macro based on the hantavirus glycoprotein structure “6ZJM” template [48]. Resulting homology models displayed three different subunit configurations (four in Configuration 1, two in Configuration 2, one in Configuration 3). These different configurations likely reflect stochastic variations in fitting or different states of the protein, as the hantavirus glycoprotein can transition through different structural variations [47]. It is unlikely that these variations reflect actual changes in protein folding, as we even observed different configurations between the near identical TULV-EST.S proteins from the Saxony transect and our reference Moravia (4 AA difference).

Pairwise structural alignments, including RMSDs and sequence identities between the homology models were calculated using MUSTANG [86]. RMSD of atomic locations across the entire protein tetramer (residue 13–1104) were compared to the RMSD of first nine AA of the Gn-subunits ectodomain (N-terminus). Only the results of the alignment for the four proteins in Configuration 1 are displayed in S10 Table, because structural alignments require identical configuration of all subunits for meaningful evaluation. Individual subunits of the three remaining proteins, when aligned, showed similar levels of low RSMDs, but randomness in the predicted configurations artificially inflates the pairwise RMSDs of the complete proteins. This problem can normally be circumvented by using the configuration of a predicted protein for all future predictions. This was not applicable for our set of proteins, as it would also artificially force the flexible N-terminal ectodomain to change shape to match the reference thus eliminating the key variation between proteins.

MD simulations of all N-terminal regions were performed using the “md.run.mcr” macro and AMBER14 force field, with a simulation duration of 500 ns [87]. Time simulation steps were set to 1.35 fsec. The simulation box was ‘Cube”-shaped and extended at least 10 Å to each side of the model (extension = 10), was filled with 0.9% NaCl and the TIP3P water model was used at physiological pH 7.4. Further settings were: temperature at 298K, pressure at 1 bar, density = 0.997, cutoff 8Å- periodic cell boundary and longrange coulomb forces (particle-mesh Ewald). The solute was kept from diffusing and crossing periodic boundaries using the CorrectDrift function [84,85]. The four flanking amino acids were fixed as anchor point during the MD simulations. To determine structural differences between the regions of interest, root mean square fluctuations (RMSF) were calculated by the “md_analyze” macro. The RMSF is the fluctuation of every heavy atom compared to the mean structure within a simulation cell. The average RMSF of the constituent atoms is used to determine the RMSF per solute residue. False positives with artificially high scores were further minimized by considering the number of structural neighbors in the model ensemble. The finished protein models can be found in the dryad repository [88].

## Supporting information

S1 Fig Phylogenetic relationships of host mtDNA from the Saxony transect. Phylogenetic analysis was based on a 458 bp fragment of Cytochrome b from 183 voles across the transect. For better display, the bottom half of the tree is displayed to the right of the upper half. Names colored in purple show reference sequences for the classification of evolutionary lineages. Bayesian posterior probabilities are included for all nodes. The scale bar on top shows evolutionary distance in substitutions per nucleotide.
(DOCX)

S2 Fig Phylogenetic relationships of partial TULV sequences from the Saxony transect. Phylogenetic analysis was based on 439 bp, 356 bp and 305 bp fragments of the S-, M- and L-segment of TULV respectively for 128 infected individuals. Names colored in purple show reference sequences for the classification of TULV clades. Bayesian posterior probabilities are included for all nodes. The scale bar on top shows evolutionary distance in substitutions per nucleotide.
(DOCX)

S3 Fig Phylogenetic relationships of amino acid sequences from complete TULV genome segments. Phylogenetic analysis was based on the complete amino acid sequence of each TULV segment with Puumala orthohantavirus (PUUV) as outgroup. Names with an asterisk show new TULV genome sequences from this study. Reassorted genomes are colored based on reassortment types. Bayesian posterior probabilities are included for all nodes. The scale bar on top shows evolutionary distance in substitutions per nucleotide. For better visualization, branches towards PUUV outgroups were truncated, indicated by double slashes through the branch. Branch lengths between PUUV and TULV are approximately 8, 10 and 4 times longer than shown for the S-, M- and L-segment respectively.
(DOCX)

S4 Fig Sliding window analyses of the TULV S-, M- and L-segments. The plots show the ratio of non-synonymous to synonymous substitutions (d_N_/d_S_, black area) and average number of nucleotide substitutions per site (D_XY_, grey area). Results are shown for the whole CDS of 36, 9 and 31 TULV-CEN.N and 20, 44, 22 TULV-EST.N S-, M- and L-segments respectively. The window size was 30 nt and step size 10 nt.
(DOCX)

S5 Fig Sliding window analysis of the M-segments of TULV-EST.N. The plot shows the ratio of non-synonymous to synonymous substitutions (d_N_/d_S_, black area) and average number of nucleotide substitutions per site (D_XY_, grey area). Results are shown for the whole CDS of the TULV M-segment between the combined cluster of 23 genomes from TULV-CEC and TULV-CEE-1 and the combined cluster of 21 genomes from TULV-CEE-2 and parental TULV-EST.N. The window size was 30 nt and step size 10 nt.
(DOCX)

S6 Fig Root mean square fluctuations (RMSF) of atoms within the N-terminal ectodomain of the glycoprotein in different TULV strains. Plots represent major TULV variants from the Saxony transect. The RMSF (Å) is shown for every atom in the first nine residues of the mature TULV glycoprotein. Residues are colored based on the CPK convention for amino acids: hydrophobic - grey, polar - magenta, acidic - red, basic - blue.
(DOCX)

S1 Table Overview of common voles analysed in this study. Voles for which genomic mtDNA or TULV RNA was sequenced and analysed are listed with their respective date and location of capture. TULV clade membership is listed for both partial and whole genome sequences. For reassorted genomes, letters denominate the clade membership of segments in the order: S-segment, M-segment, L-segment. C: TULV-CEN.N, E: TULV-EST.N.
(DOCX)

S2 Table Reference sequences for phylogenetic clustering of TULV. Sequences were obtainted from the NCBI database for the assignment of phylogenetic clusters to large-scale evolutionary clades TULV.
(DOCX)

S3 Table Concordance of cline widths and centers. P-values are given for pairwise likelihood ratio tests for concordance between respective geographic clines. Only for the comparison between L- and S-segment was concordance of cline widths and centers not rejected.
(DOCX)

S4 Table Overview of phylogenetic clade affiliation and reassortment types of TULV in the Saxony transect. Reassortants are separated by individual reassortment types. For reassortant types letters denominate the clade membership of segments in the order: S-segment, M-segment, L-segment. C: TULV-CEN.N, E: TULV-EST.N.
(DOCX)

S5 Table Coverage statistics for all sequenced TULV genomes. The table shows metrics for each genomic segment of each TULV genome separately and combined for the whole genome. All genomes covered 98.2% to 99.8% of the full sequence of the reference genome Moravia [19], with 98.2% of all sites covered by at least 3 reads and 95.8% by at least 20. For the four genomes with double infections, a second row shows the statistics for the assembly of TULV-CEN.N. Numbers in red indicate genomic segments that were absent. Reads for segments in red would assemble the same TULV-EST.N genome, both when mapped against TULV-EST.N and TULV-CEN.N, albeit with a notably lower read count for the latter. The table shows the complete length of the assembled segment, total count of assembled reads, average read depth, the percentage of sites with a read depth of at least 3 and the percentage of all sites with a read depth of at least 20.
(DOCX)

S6 Table Divergence of TULV clades for different genome segments. The table shows nucleotide diversity of whole genomic segments within the TULV-CEN.N and TULV-EST.N clades and the net nucleotide divergence between them. S-Segment: N-TULV-CEN.N = 36, N-TULV-EST.N = 20; M-Segment: N-TULV-CEN.N = 9. N-TULV-EST.N = 44, L-Segment: N-TULV-CEN.N = 31, N-TULV-EST.N = 22. Results are shown for the coding nucleotide sequence (nt), the amino acid sequence (AA), and d_N_/d_S_.
(DOCX)

S7 Table Phylogenetic dating of TULV S-, M- & L-segments with BEAST. Times to most recent common ancestor in years were estimated based on a substitution rate of 1.51 × 10^−3^ substitutions per site per year for the full phylogeny, TULV-CEN.N and TULV-EST.N, as well as for TULV-CEC and the combined cluster of TULV-CEC & TULV-CEE-1. Brackets indicate the 95% confidence intervals.
(DOCX)

S8 Table Results of the analysis for signatures of selection with the branch-site model for the TULV S-, M- & L-segments. We used CodeML to perform branch site (BrS) tests. In two separate analyses, we partitioned the data into the TULV-CEN.N and TULV-EST.N clades for all segments and the cluster of TULV-CEC & CEE-1 combined and the cluster of TULV-CEE-2 and parental TULV-EST.N combined. Bayes empirical bayes inference was used to detect codons under positive selection, which are indicated with their posterior probability in brackets. Abbreviations in the table read as follows: np, number of model parameters; lnL, model likelihood; κ, transition to transversion ratio; ω, d_N_/d_S_ ratio, LRT, the *D* value of a likelihood ratio test; *p*-value, the *p*-value derived from a χ^2^ distribution with 1 degree of freedom.
(DOCX)

S9 Table Results of the analysis for signatures of selection with the MEME and FUBAR methods in HYPHY for the TULV S-, M- & L-segments. All segments from the TULV-CEN.N and TULV-EST.N genomes of the Saxony transect were analysed individually. Additionally, we also analysed the M-segment of the cluster of TULV-CEC & CEE-1 combined and the cluster of TULV-CEE-2 and parental TULV-EST.N combined for evidence of positive or purifying selection within the clade. Positively selected codons are indicated with the posterior probability *P* (> 0.85) for FUBAR or a *p*-value (< 0.1) for MEME.
(DOCX)

S10 Table Root mean square deviations RMSDs (Å) of the N-terminal ectodomains of the TULV glycoprotein compared to complete homology models. Distances were averaged across both the N-terminal ectodomain only and the full length of the protein (in brackets). Only four out seven protein models with identical subunit rotational symmetry were included in the evaluation of structural alignments.
(DOCX)
