# Supplementary material for: Host hybridization enabled the emergence of a reassorted hantavirus lineage
Source: PLoS Pathog. 2026 Jul 28;22(7):e1014458. doi: 10.1371/journal.ppat.1014458 (PMC13411931; doi:10.1371/journal.ppat.1014458)
Supplement: S1 Fig — Phylogenetic analysis was based on a 458 bp fragment of Cytochrome b from 183 voles across the transect. For better display, the bottom half of the tree is displayed to the right of the upper half. Names colored in purple show reference sequences for the classification of evolutionary lineages. Bayesian posterior probabilities are included for all nodes. The scale bar on top shows evolutionary distance in substitutions per nucleotide. (DOCX) [file ppat.1014458.s001.docx]

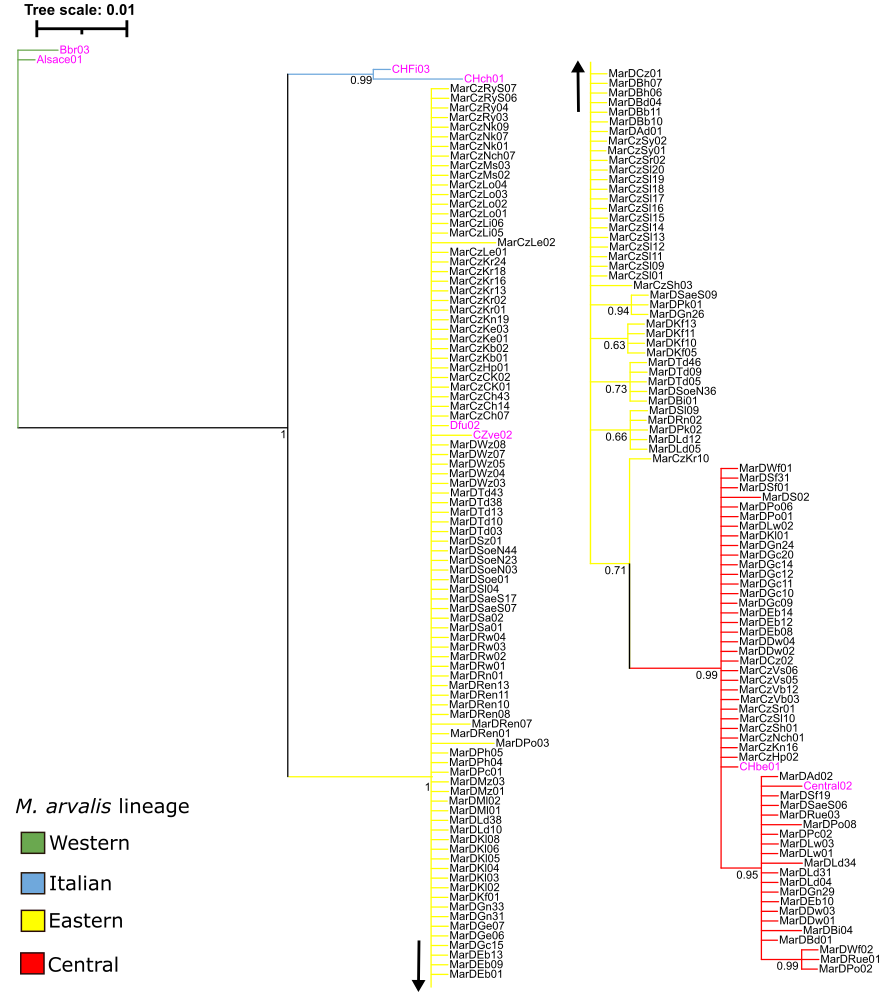


**S1 Fig: Phylogenetic relationships of host mtDNA from the Saxony transect.** Phylogenetic analysis was based on a 458 bp fragment of Cytochrome b from 183 voles across the transect. For better display, the bottom half of the tree is displayed to the right of the upper half. Names colored in purple show reference sequences for the classification of evolutionary lineages. Bayesian posterior probabilities are included for all nodes. The scale bar on top shows evolutionary distance in substitutions per nucleotide.
