# Supplementary material for: Host hybridization enabled the emergence of a reassorted hantavirus lineage
Source: PLoS Pathog. 2026 Jul 28;22(7):e1014458. doi: 10.1371/journal.ppat.1014458 (PMC13411931; doi:10.1371/journal.ppat.1014458)
Supplement: S2 Fig — Phylogenetic analysis was based on 439 bp, 356 bp and 305 bp fragments of the S-, M- and L-segment of TULV respectively for 128 infected individuals. Names colored in purple show reference sequences for the classification of TULV clades. Bayesian posterior probabilities are included for all nodes. The scale bar on top shows evolutionary distance in substitutions per nucleotide. (DOCX) [file ppat.1014458.s002.docx]

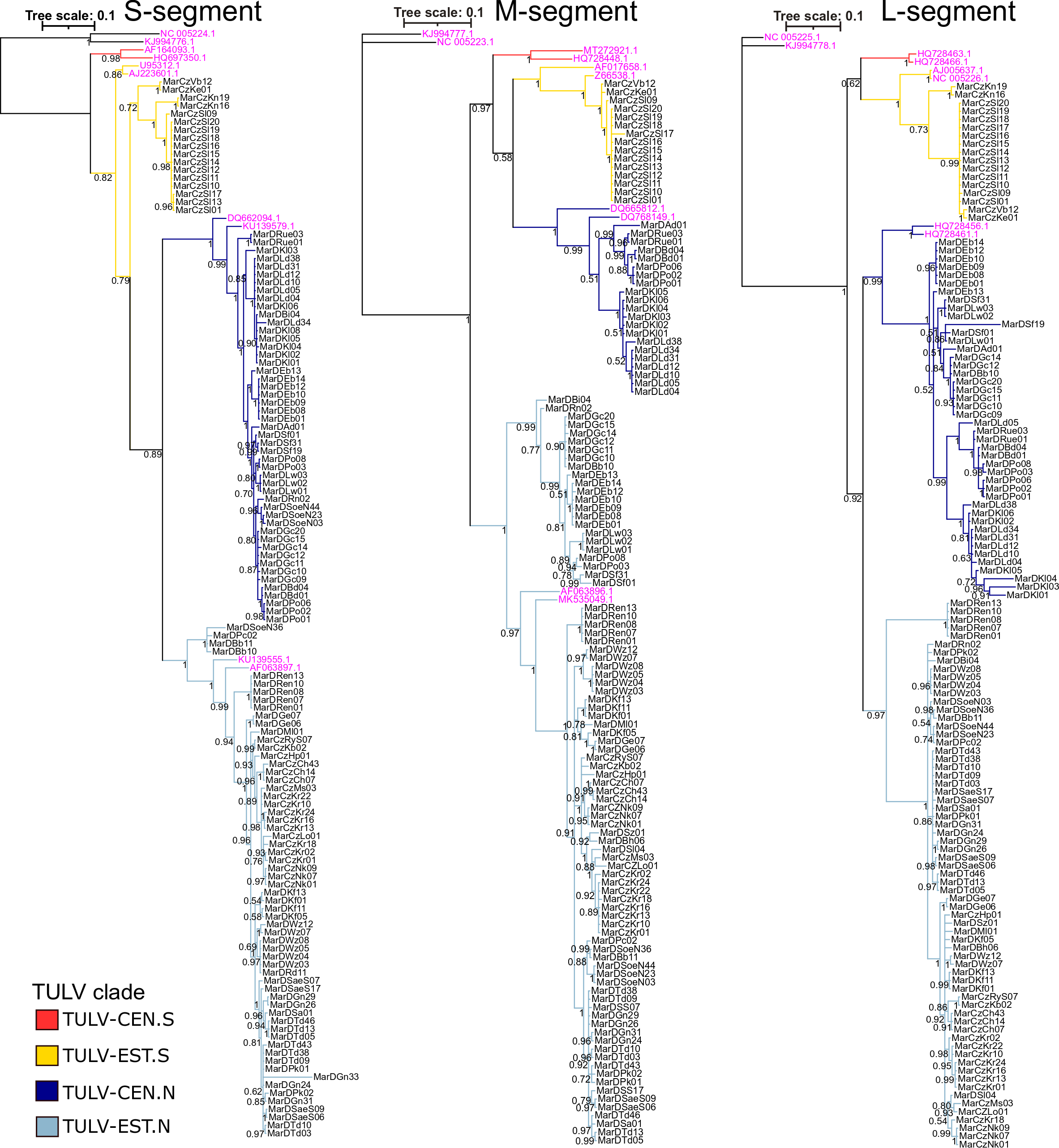


**S2 Fig: Phylogenetic relationships of partial TULV sequences from the Saxony transect.** Phylogenetic analysis was based on 439 bp, 356 bp and 305 bp fragments of the S-, M- and L-segment of TULV respectively for 128 infected individuals. Names colored in purple show reference sequences for the classification of TULV clades. Bayesian posterior probabilities are included for all nodes. The scale bar on top shows evolutionary distance in substitutions per nucleotide.
