# Supplementary material for: Host hybridization enabled the emergence of a reassorted hantavirus lineage
Source: PLoS Pathog. 2026 Jul 28;22(7):e1014458. doi: 10.1371/journal.ppat.1014458 (PMC13411931; doi:10.1371/journal.ppat.1014458)
Supplement: S3 Fig — Phylogenetic analysis was based on the complete amino acid sequence of each TULV segment with Puumala orthohantavirus (PUUV) as outgroup. Names with an asterisk show new TULV genome sequences from this study. Reassorted genomes are colored based on reassortment types. Bayesian posterior probabilities are included for all nodes. The scale bar on top shows evolutionary distance in substitutions per nucleotide. For better visualization, branches towards PUUV outgroups were truncated, indicated by double slashes through the branch. Branch lengths between PUUV and TULV are approximately 8, 10 and 4 times longer than shown for the S-, M- and L-segment respectively. (DOCX) [file ppat.1014458.s003.docx]

**
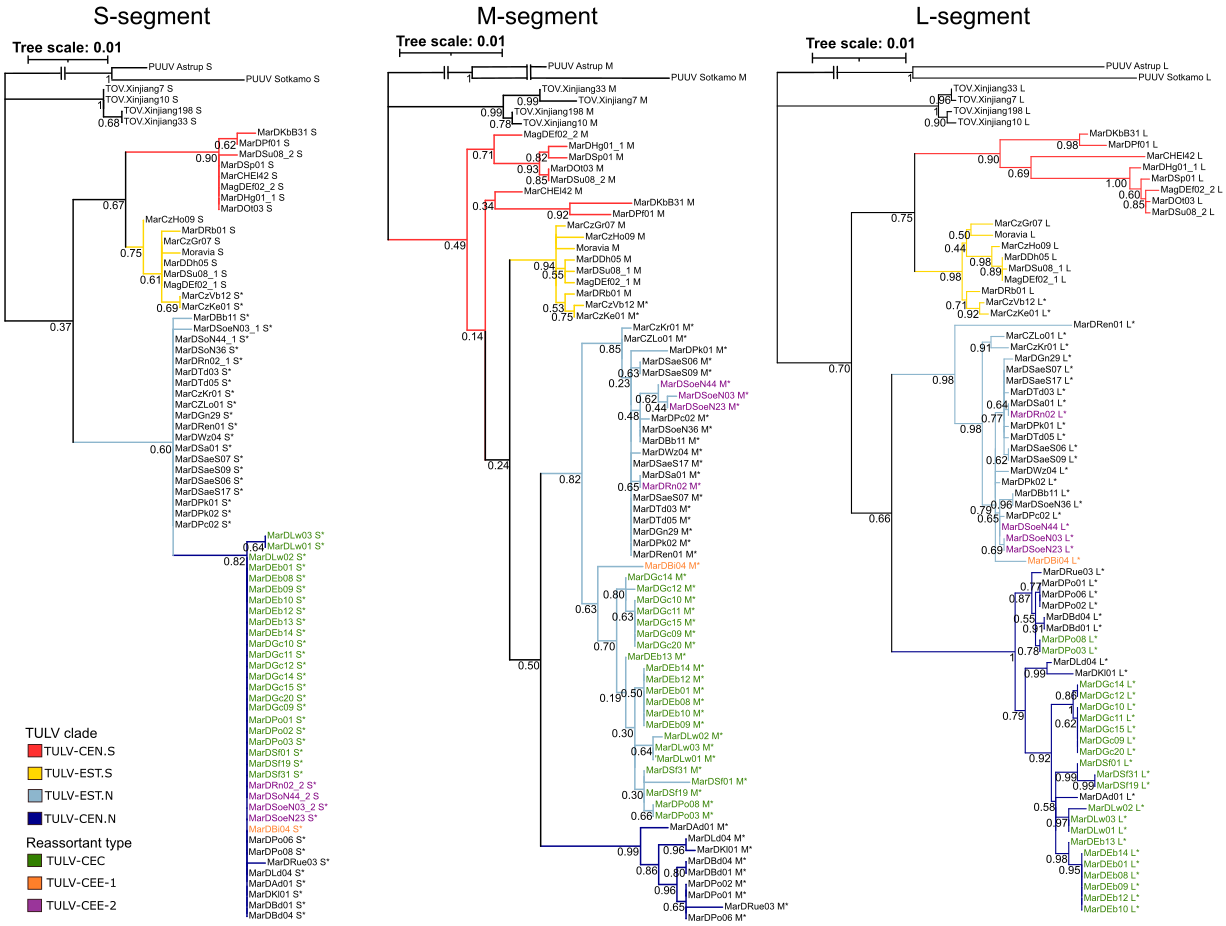
**

**S3 Fig: Phylogenetic relationships of amino acid sequences from complete TULV genome segments.** Phylogenetic analysis was based on the complete amino acid sequence of each TULV segment with Puumala orthohantavirus (PUUV) as outgroup. Names with an asterisk show new TULV genome sequences from this study. Reassorted genomes are colored based on reassortment types. Bayesian posterior probabilities are included for all nodes. The scale bar on top shows evolutionary distance in substitutions per nucleotide. For better visualization, branches towards PUUV outgroups were truncated, indicated by double slashes through the branch. Branch lengths between PUUV and TULV are approximately 8, 10 and 4 times longer than shown for the S-, M- and L-segment respectively.
