# Supplementary material for: Host hybridization enabled the emergence of a reassorted hantavirus lineage
Source: PLoS Pathog. 2026 Jul 28;22(7):e1014458. doi: 10.1371/journal.ppat.1014458 (PMC13411931; doi:10.1371/journal.ppat.1014458)
Supplement: S4 Fig — The plots show the ratio of non-synonymous to synonymous substitutions (dN/dS, black area) and average number of nucleotide substitutions per site (DXY, grey area). Results are shown for the whole CDS of 36, 9 and 31 TULV-CEN.N and 20, 44, 22 TULV-EST.N S-, M- and L-segments respectively. The window size was 30 nt and step size 10 nt. (DOCX) [file ppat.1014458.s004.docx]

**
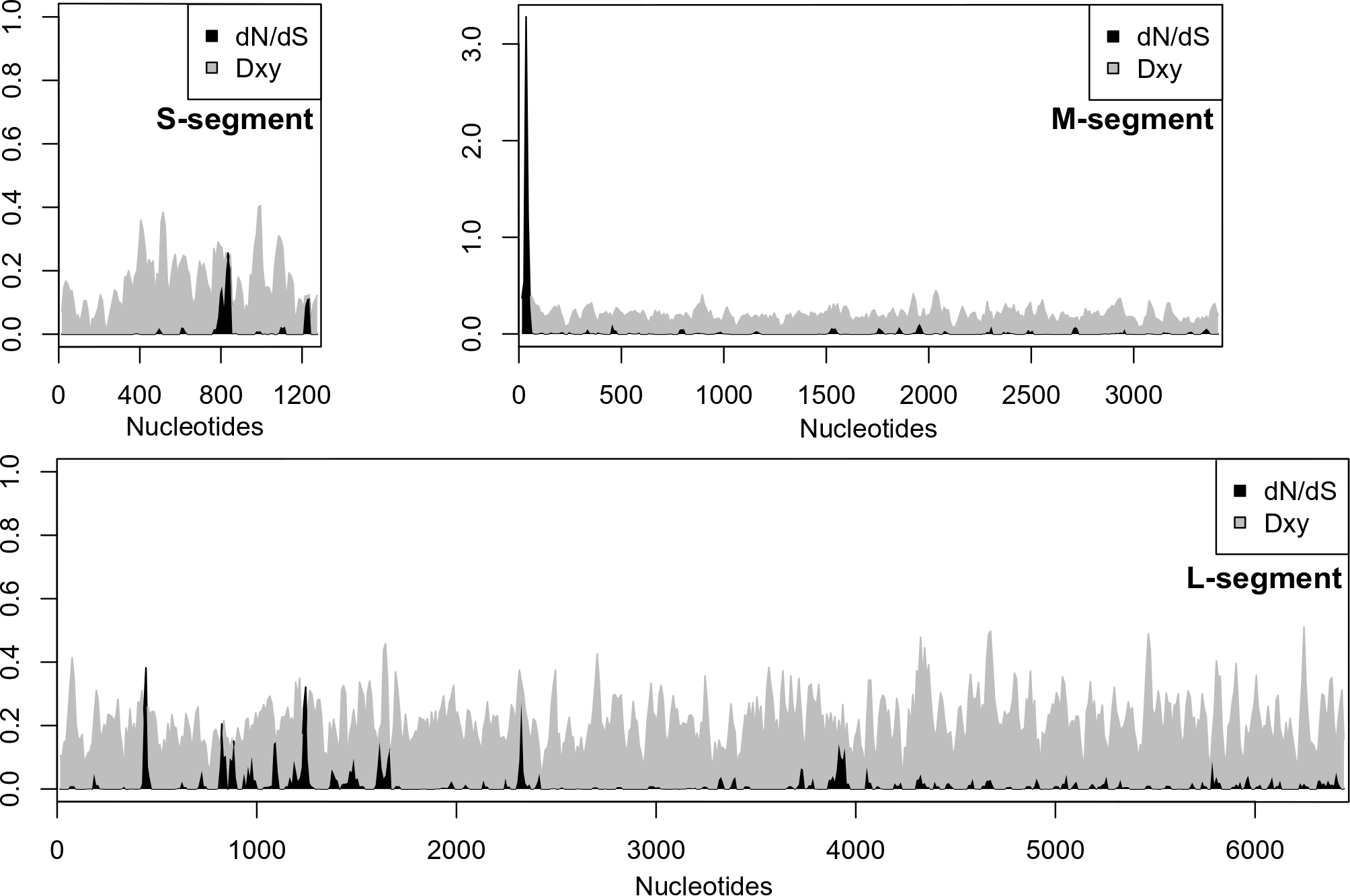
**

**S4 Fig: Sliding window analyses of the TULV S-, M- and L-segments**. The plots show the ratio of non-synonymous to synonymous substitutions (d_N_/d_S_, black area) and average number of nucleotide substitutions per site (D_XY_, grey area). Results are shown for the whole CDS of 36, 9 and 31 TULV-CEN.N and 20, 44, 22 TULV-EST.N S-, M- and L-segments respectively. The window size was 30 nt and step size 10 nt.
