# Supplementary material for: Host hybridization enabled the emergence of a reassorted hantavirus lineage
Source: PLoS Pathog. 2026 Jul 28;22(7):e1014458. doi: 10.1371/journal.ppat.1014458 (PMC13411931; doi:10.1371/journal.ppat.1014458)
Supplement: S5 Fig — The plot shows the ratio of non-synonymous to synonymous substitutions (dN/dS, black area) and average number of nucleotide substitutions per site (DXY, grey area). Results are shown for the whole CDS of the TULV M-segment between the combined cluster of 23 genomes from TULV-CEC and TULV-CEE-1 and the combined cluster of 21 genomes from TULV-CEE-2 and parental TULV-EST.N. The window size was 30 nt and step size 10 nt. (DOCX) [file ppat.1014458.s005.docx]

**
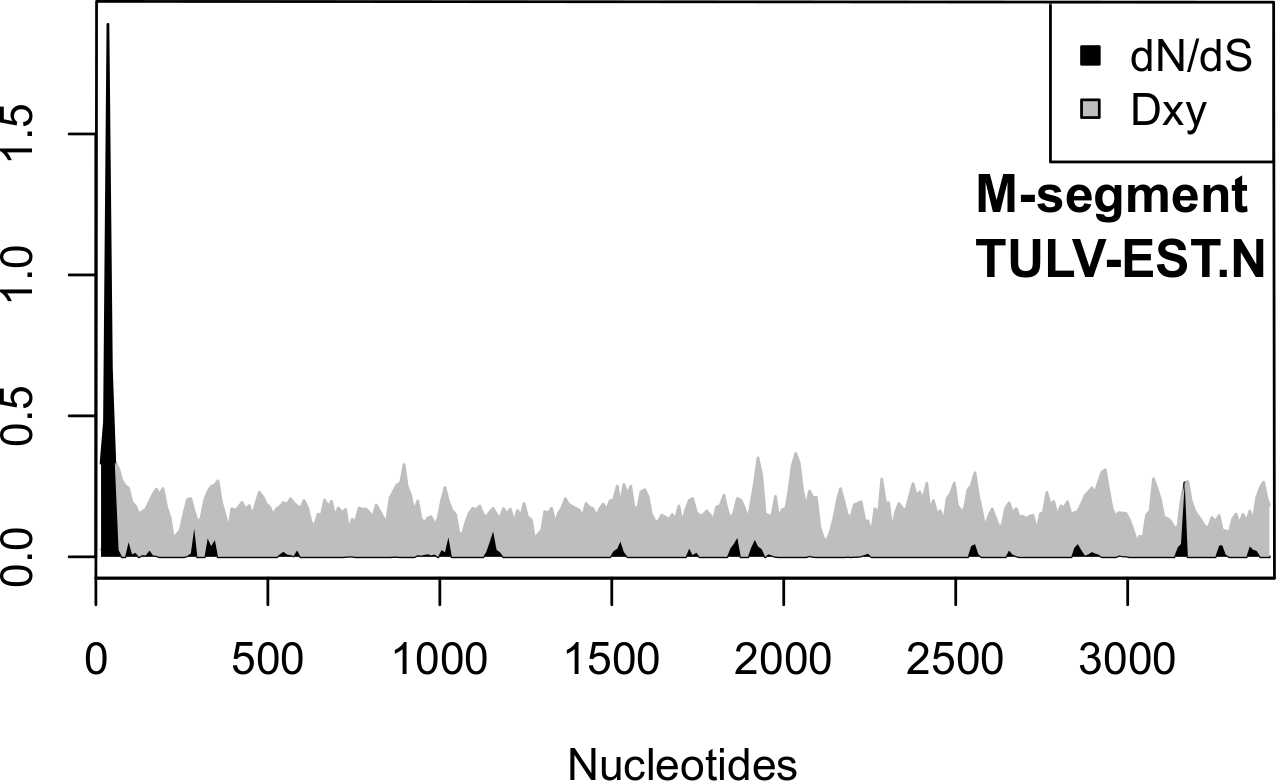
**

**S5 Fig: Sliding window analysis of the M-segments of TULV-EST.N. The plot shows the ratio of non-synonymous to synonymous substitutions (d_N_/d_S_, black area) and average number of nucleotide substitutions per site (D_XY_, grey area). Results are shown for the whole CDS of the TULV M-segment between the combined cluster of 23 genomes from TULV-CEC and TULV-CEE-1 and the combined cluster of 21 genomes from TULV-CEE-2 and parental TULV-EST.N. The window size was 30 nt and step size 10 nt.**
