# Supplementary material for: Host hybridization enabled the emergence of a reassorted hantavirus lineage
Source: PLoS Pathog. 2026 Jul 28;22(7):e1014458. doi: 10.1371/journal.ppat.1014458 (PMC13411931; doi:10.1371/journal.ppat.1014458)
Supplement: S6 Fig — Plots represent major TULV variants from the Saxony transect. The RMSF (Å) is shown for every atom in the first nine residues of the mature TULV glycoprotein. Residues are colored based on the CPK convention for amino acids: hydrophobic - grey, polar - magenta, acidic - red, basic - blue. (DOCX) [file ppat.1014458.s006.docx]

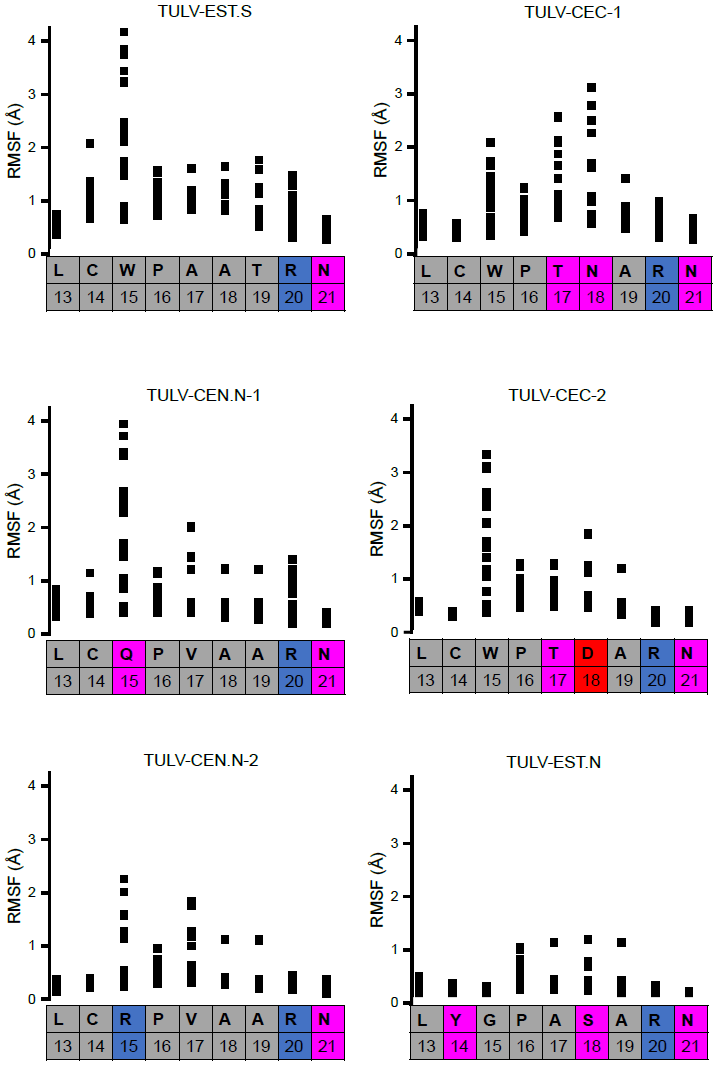


**S6 Fig: Root mean square fluctuations (RMSF) of atoms within the N-terminal ectodomain of the glycoprotein in different TULV strains.** Plots represent major TULV variants from the Saxony transect. The RMSF (Å) is shown for every atom in the first nine residues of the mature TULV glycoprotein. Residues are colored based on the CPK convention for amino acids: hydrophobic - grey, polar - magenta, acidic - red, basic - blue.
