# Supplementary material for: Host hybridization enabled the emergence of a reassorted hantavirus lineage
Source: PLoS Pathog. 2026 Jul 28;22(7):e1014458. doi: 10.1371/journal.ppat.1014458 (PMC13411931; doi:10.1371/journal.ppat.1014458)
Supplement: S1 Table — Voles for which genomic mtDNA or TULV RNA was sequenced and analysed are listed with their respective date and location of capture. TULV clade membership is listed for both partial and whole genome sequences. For reassorted genomes, letters denominate the clade membership of segments in the order: S-segment, M-segment, L-segment. C: TULV-CEN.N, E: TULV-EST.N. (DOCX) [file ppat.1014458.s007.docx]

**S1 Table: Overview of common voles analysed in this study.** Voles for which genomic mtDNA or TULV RNA was sequenced and analysed are listed with their respective date and location of capture. TULV clade membership is listed for both partial and whole genome sequences. For reassorted genomes, letters denominate the clade membership of segments in the order: S-segment, M-segment, L-segment. C: TULV-CEN.N, E: TULV-EST.N.
