# Supplementary material for: Host hybridization enabled the emergence of a reassorted hantavirus lineage
Source: PLoS Pathog. 2026 Jul 28;22(7):e1014458. doi: 10.1371/journal.ppat.1014458 (PMC13411931; doi:10.1371/journal.ppat.1014458)
Supplement: S2 Table — Sequences were obtainted from the NCBI database for the assignment of phylogenetic clusters to large-scale evolutionary clades TULV. (DOCX) [file ppat.1014458.s008.docx]

**S2 Table:** **Reference sequences for phylogenetic clustering of TULV.** Sequences were obtainted from the NCBI database for the assignment of phylogenetic clusters to large-scale evolutionary clades TULV.

| Segment | Accession number | Clade |
| --- | --- | --- |
| S | NC005224 | PUUV |
| S | KJ994776 | PUUV |
| S | AF063897 | Eastern North |
| S | KU139555 | Eastern North |
| S | KU139579 | Central North |
| S | DQ662094 | Central North |
| S | AF164093 | Central South |
| S | HQ697350 | Central South |
| S | AJ223601 | Eastern South |
| S | U95312 | Eastern South |
| M | NC005223 | PUUV |
| M | KJ994777 | PUUV |
| M | AF063896 | Eastern North |
| M | MK535049 | Eastern North |
| M | DQ665812 | Central North |
| M | DQ768149 | Central North |
| M | MK386151 | Central South |
| M | MT272921 | Central South |
| M | AF017658 | Eastern South |
| M | Z66538 | Eastern South |
| L | KJ994778 | PUUV |
| L | NC005225 | PUUV |
| L | HQ728461 | Central North |
| L | HQ728456 | Central North |
| L | HQ728466 | Central South |
| L | HQ728463 | Central South |
| L | NC005226 | Eastern South |
| L | AJ005637 | Eastern South |
