# Supplementary material for: Host hybridization enabled the emergence of a reassorted hantavirus lineage
Source: PLoS Pathog. 2026 Jul 28;22(7):e1014458. doi: 10.1371/journal.ppat.1014458 (PMC13411931; doi:10.1371/journal.ppat.1014458)
Supplement: S3 Table — P-values are given for pairwise likelihood ratio tests for concordance between respective geographic clines. Only for the comparison between L- and S-segment was concordance of cline widths and centers not rejected. (DOCX) [file ppat.1014458.s009.docx]

**S3 Table: Concordance of cline widths and centers.** P-values are given for pairwise likelihood ratio tests for concordance between respective geographic clines. Only for the comparison between L- and S-segment was concordance of cline widths and centers not rejected.

|  | S-segment | M-segment | L-segment |
| --- | --- | --- | --- |
| mtDNA | <0.001 | 0.007 | <0.001 |
| S-segment |  | <0.001 | 0.36 |
| M-segment |  |  | <0.001 |
