# Supplementary material for: Host hybridization enabled the emergence of a reassorted hantavirus lineage
Source: PLoS Pathog. 2026 Jul 28;22(7):e1014458. doi: 10.1371/journal.ppat.1014458 (PMC13411931; doi:10.1371/journal.ppat.1014458)
Supplement: S4 Table — Reassortants are separated by individual reassortment types. For reassortant types letters denominate the clade membership of segments in the order: S-segment, M-segment, L-segment. C: TULV-CEN.N, E: TULV-EST.N. (DOCX) [file ppat.1014458.s010.docx]

**S4 Table: Overview of phylogenetic clade affiliation and reassortment types of TULV in the Saxony transect.** Reassortants are separated by individual reassortment types. For reassortant types letters denominate the clade membership of segments in the order: S-segment, M-segment, L-segment. C: TULV-CEN.N, E: TULV-EST.N.

|  | Genomes |
| --- | --- |
| TULV-CEN.N | 9 |
| TULV-EST.N | 17 |
| TULV-EST.S | 2 |
| TULV-CEC | 22 |
| TULV-CEE-1 | 1 |
| TULV-CEE-2 | 4 |
| Total | 55 |
