# Supplementary material for: Host hybridization enabled the emergence of a reassorted hantavirus lineage
Source: PLoS Pathog. 2026 Jul 28;22(7):e1014458. doi: 10.1371/journal.ppat.1014458 (PMC13411931; doi:10.1371/journal.ppat.1014458)
Supplement: S5 Table — The table shows metrics for each genomic segment of each TULV genome separately and combined for the whole genome. All genomes covered 98.2% to 99.8% of the full sequence of the reference genome Moravia [19], with 98.2% of all sites covered by at least 3 reads and 95.8% by at least 20. For the four genomes with double infections, a second row shows the statistics for the assembly of TULV-CEN.N. Numbers in red indicate genomic segments that were absent. Reads for segments in red would assemble the same TULV-EST.N genome, both when mapped against TULV-EST.N and TULV-CEN.N, albeit with a notably lower read count for the latter. The table shows the complete length of the assembled segment, total count of assembled reads, average read depth, the percentage of sites with a read depth of at least 3 and the percentage of all sites with a read depth of at least 20. (DOCX) [file ppat.1014458.s011.docx]

**S5** **Table: Coverage statistics for all sequenced TULV genomes.** The table shows metrics for each genomic segment of each TULV genome separately and combined for the whole genome. All genomes covered 98.2% to 99.8% of the full sequence of the reference genome Moravia (19), with 98.2% of all sites covered by at least 3 reads and 95.8% by at least 20. For the four genomes with double infections, a second row shows the stats for the assembly of TULV-CEN.N. Numbers in red indicate genomic segments that were absent. Reads for segments in red would assemble the same TULV-EST.N genome, both when mapped against TULV-EST.N and TULV-CEN.N, albeit with a notably lower read count for the latter. The table shows the complete length of the assembled segment, total count of assembled reads, average read depth, the percentage of sites with a read depth of at least 3 and the percentage of all sites with a read depth of at least 20.
