# Supplementary material for: Host hybridization enabled the emergence of a reassorted hantavirus lineage
Source: PLoS Pathog. 2026 Jul 28;22(7):e1014458. doi: 10.1371/journal.ppat.1014458 (PMC13411931; doi:10.1371/journal.ppat.1014458)
Supplement: S6 Table — The table shows nucleotide diversity of whole genomic segments within the TULV-CEN.N and TULV-EST.N clades and the net nucleotide divergence between them. S-Segment: N-TULV-CEN.N = 36, N-TULV-EST.N = 20; M-Segment: N-TULV-CEN.N = 9. N-TULV-EST.N = 44, L-Segment: N-TULV-CEN.N = 31, N-TULV-EST.N = 22. Results are shown for the coding nucleotide sequence (nt), the amino acid sequence (AA), and dN/dS. (DOCX) [file ppat.1014458.s012.docx]

**S6** **Table: Divergence of TULV clades for different genome segments.** The table shows nucleotide diversity of whole genomic segments within the TULV-CEN.N and TULV-EST.N clades and the net nucleotide divergence between them. S-Segment: N-TULV-CEN.N=36, N-TULV-EST.N=20; M-Segment: N-TULV-CEN.N=9. N-TULV-EST.N=44, L-Segment: N-TULV-CEN.N=31, N-TULV-EST.N=22. Results are shown for the coding nucleotide sequence (nt), the amino acid sequence (AA), and d_N_/d_S_.

|  |  | S-segment | M-segment | L-segment |
| --- | --- | --- | --- | --- |
|  | **Length** | 1290 | 3423 | 6459 |
| TULV-CEN.N | **nt** | 1.82% | 4.01% | 4.16% |
|  | **AA** | 0.04% | 0.47% | 0.66% |
|  | **dN/dS** | 0.002 | 0.012 | 0.016 |
| TULV-EST.N | **nt** | 5.40% | 7.71% | 3.47% |
|  | **AA** | 0.05% | 0.63% | 0.41% |
|  | **dN/dS** | 0.006 | 0.014 | 0.013 |
| Between clades | **nt** | 13.81% | 15.37% | 18.67% |
|  | **AA** | 0.97% | 1.96% | 2.74% |
|  | **dN/dS** | 0.011 | 0.012 | 0.011 |
