# Supplementary material for: Host hybridization enabled the emergence of a reassorted hantavirus lineage
Source: PLoS Pathog. 2026 Jul 28;22(7):e1014458. doi: 10.1371/journal.ppat.1014458 (PMC13411931; doi:10.1371/journal.ppat.1014458)
Supplement: S7 Table — Times to most recent common ancestor in years were estimated based on a substitution rate of 1.51 × 10^−3^ substitutions per site per year for the full phylogeny, TULV-CEN.N and TULV-EST.N, as well as for TULV-CEC and the combined cluster of TULV-CEC & TULV-CEE-1. Brackets indicate the 95% confidence intervals. (DOCX) [file ppat.1014458.s013.docx]

**S7** **Table: Phylogenetic dating of TULV S-, M- & L-segments with** **BEAST**. Times to most recent common ancestor in years were estimated based on a substitution rate of 1.51 × 10^−3^ substitutions per site per year for the full phylogeny, TULV-CEN.N and TULV-EST.N, as well as for TULV-CEC and the combined cluster of TULV-CEC & TULV-CEE-1. Brackets indicate the 95% confidence intervals.

|  | S-segment | M-segment | L-segment |
| --- | --- | --- | --- |
| All sequences | 112 (42.2 – 191.4) | 177.9 (70.7 – 329.5) | 214.2 (92.7 – 388.2) |
| TULV-CEN.N | 29 (11.8 – 50.4) | 40.8 (14.1 – 76.1) | 48.2 (21.5 – 78.9) |
| TULV-EST.N | 58.2 (25 – 100) | 104.6 (47.7 – 181) | 114.2 (43.7 – 182.6) |
| TULV-CEC & TULV-CEE-1 | 13.5 (7.3 – 21.3) | 33.7 (15.9 – 57.5) | 214.2 (92.7 – 388.2) |
| TULV-CEC | 9 (5.1 – 13.6) | 16.6 (8.7 – 25.6) | 32.6 (17.3 – 48.7) |
