# Supplementary material for: Host hybridization enabled the emergence of a reassorted hantavirus lineage
Source: PLoS Pathog. 2026 Jul 28;22(7):e1014458. doi: 10.1371/journal.ppat.1014458 (PMC13411931; doi:10.1371/journal.ppat.1014458)
Supplement: S8 Table — We used CodeML to perform branch site (BrS) tests. In two separate analyses, we partitioned the data into the TULV-CEN.N and TULV-EST.N clades for all segments and the cluster of TULV-CEC & CEE-1 combined and the cluster of TULV-CEE-2 and parental TULV-EST.N combined. Bayes empirical bayes inference was used to detect codons under positive selection, which are indicated with their posterior probability in brackets. Abbreviations in the table read as follows: np, number of model parameters; lnL, model likelihood; κ, transition to transversion ratio; ω, dN/dS ratio, LRT, the D value of a likelihood ratio test; p-value, the p-value derived from a χ2 distribution with 1 degree of freedom. (DOCX) [file ppat.1014458.s014.docx]

**S8** **Table: Results of the analysis for signatures of selection with the branch-site model for the TULV S-, M- & L-segments.** We used CodeML to perform branch site (BrS) tests. In two separate analyses, we partitioned the data into the TULV-CEN.N and TULV-EST.N clades for all segments and the cluster of TULV-CEC & CEE-1 combined and the cluster of TULV-CEE-2 and parental TULV-EST.N combined. Bayes empirical bayes inference was used to detect codons under positive selection, which are indicated with their posterior probability in brackets. Abbreviations in the table read as follows: np, number of model parameters; lnL, model likelihood; κ, transition to transversion ratio; ω, d_N_/d_S_ ratio, LRT, the *D* value of a likelihood ratio test; *p*-value, the *p*-value derived from a χ^2^ distribution with 1 degree of freedom.

| Segment | Partition | Model | np | lnL | *κ* | *ω*0 | *ω*1 | *ω*2a | *ω*2b | LRT | *p*-value | Sites (*P* > 0.75) |
| --- | --- | --- | --- | --- | --- | --- | --- | --- | --- | --- | --- | --- |
| S | TULV-EST.N | BrS_null | 113 | -4168.99 | 6.107 | BG: 0.00245 | BG: 1 | BG: 0.00245 | BG: 1 |  |  |  |
|  |  |  |  |  |  | FG: 0.00245 | FG: 1 | FG: 1 | FG: 1 |  |  |  |
|  |  |  |  |  |  | 98.699% | 0.212% | 1.086% | 0.002% |  |  |  |
|  |  | BrS | 114 | -4168.99 | 6.107 | BG: 0.00245 | BG: 1 | BG: 0.00245 | BG: 1 | 0 | 1 |  |
|  |  |  |  |  |  | FG: 0.00245 | FG: 1 | FG: 1 | FG: 1 |  |  |  |
|  |  |  |  |  |  | 98.699% | 0.212% | 1.086% | 0.002% |  |  |  |
|  | TULV-CEN.N | BrS_null | 113 | -4169.94 | 6.023 | BG: 0.00320 | BG: 1 | BG: 0.00320 | BG: 1 |  |  |  |
|  |  |  |  |  |  | FG: 0.00320 | FG: 1 | FG: 1 | FG: 1 |  |  |  |
|  |  |  |  |  |  | 99.813% | 0.187% | 0.000% | 0.000% |  |  |  |
|  |  | BrS | 114 | -4169.94 | 6.023 | BG: 0.00320 | BG: 1 | BG: 0.00320 | BG: 1 | 0 | 1 |  |
|  |  |  |  |  |  | FG: 0.00320 | FG: 1 | FG: 17.71850 | FG: 17.71850 |  |  |  |
|  |  |  |  |  |  | 99.813% | 0.187% | 0.000% | 0.000% |  |  |  |
| M | TULV-EST.N | BrS_null | 107 | -12890.31 | 8.667 | BG: 0.00286 | BG: 1 | BG: 0.00286 | BG: 1 |  |  | 56 (0.862) |
|  |  |  |  |  |  | FG: 0.00286 | FG: 1 | FG: 1 | FG: 1 |  |  | 656 (0.827) |
|  |  |  |  |  |  | 98.116% | 0.817% | 1.058% | 0.009% |  |  |  |
|  |  | BrS | 108 | -12890.31 | 8.667 | BG: 0.00286 | BG: 1 | BG: 0.00286 | BG: 1 | 2E-06 | 1 |  |
|  |  |  |  |  |  | FG: 0.00286 | FG: 1 | FG: 1 | FG: 1 |  |  |  |
|  |  |  |  |  |  | 98.116% | 0.817% | 1.058% | 0.009% |  |  |  |
|  | TULV-CEN.N | BrS_null | 107 | -12889.50 | 8.675 | BG: 0.00272 | BG: 1 | BG: 0.00272 | BG: 1 |  |  |  |
|  |  |  |  |  |  | FG: 0.00272 | FG: 1 | FG: 1 | FG: 1 |  |  |  |
|  |  |  |  |  |  | 97.095% | 0.963% | 1.924% | 0.019% |  |  |  |
|  |  | BrS | 108 | -12889.50 | 8.675 | BG: 0.00272 | BG: 1 | BG: 0.00272 | BG: 1 | 0 | 0.999 |  |
|  |  |  |  |  |  | FG: 0.00272 | FG: 1 | FG: 1 | FG: 1 |  |  |  |
|  |  |  |  |  |  | 97.095% | 0.963% | 1.923% | 0.019% |  |  |  |
| L | TULV-EST.N | BrS_null | 107 | -23910.40 | 6.501 | BG: 0.00472 | BG: 1 | BG: 0.00472 | BG: 1 |  |  |  |
|  |  |  |  |  |  | FG: 0.00472 | FG: 1 | FG: 1 | FG: 1 |  |  |  |
|  |  |  |  |  |  | 99.053% | 0.577% | 0.368% | 0.002% |  |  |  |
|  |  | BrS | 108 | -23910.402 | 6.501 | BG: 0.00472 | BG: 1 | BG: 0.00472 | BG: 1 | -4E-06 | 0.999 |  |
|  |  |  |  |  |  | FG: 0.00472 | FG: 1 | FG: 1 | FG: 1 |  |  |  |
|  |  |  |  |  |  | 99.054% | 0.577% | 1.524% | 0.002% |  |  |  |
|  | TULV-CEN.N | BrS_null | 107 | -23911.33 | 6.513 | BG: 0.00480 | BG: 1 | BG: 0.00480 | BG: 1 |  |  |  |
|  |  |  |  |  |  | FG: 0.00480 | FG: 1 | FG: 1 | FG: 1 |  |  |  |
|  |  |  |  |  |  | 98.470% | 0.583% | 0.941% | 0.006% |  |  |  |
|  |  | BrS | 108 | -23911.33 | 6.513 | BG: 0.00480 | BG: 1 | BG: 0.00480 | BG: 1 | 0.00E+00 | 0.990 |  |
|  |  |  |  |  |  | FG: 0.00480 | FG: 1 | FG: 1.12096 | FG: 1.12096 |  |  |  |
|  |  |  |  |  |  | 98.470% | 0.583% | 0.941% | 0.006% |  |  |  |
|  |  |  |  |  |  |  |  |  |  |  |  |  |
| M | TULV-CEE & CEC-1 | BrS_null | 89 | -10168.06 | 7.723 | BG: 0.00297 | BG: 1 | BG: 0.00297 | BG: 1 |  |  |  |
|  |  |  |  |  |  | FG: 0.00297 | FG: 1 | FG: 1 | FG: 1 |  |  |  |
|  |  |  |  |  |  | 97.123% | 1.401% | 1.455% | 0.021% |  |  |  |
|  |  | BrS | 90 | -10168.06 | 7.723 | BG: 0.00297 | BG: 1 | BG: 0.00297 | BG: 1 | 2E-06 | 1 |  |
|  |  |  |  |  |  | FG: 0.00297 | FG: 1 | FG: 1 | FG: 1 |  |  |  |
|  |  |  |  |  |  | 97.122% | 1.401% | 1.455% | 0.021% |  |  |  |
|  | TULV-EST.N & CEC-2 | BrS_null | 89 | -9915.62 | 9.567 | BG: 0.00360 | BG: 1 | BG: 0.00360 | BG: 1 |  |  |  |
|  |  |  |  |  |  | FG: 0.00360 | FG: 1 | FG: 1 | FG: 1 |  |  |  |
|  |  |  |  |  |  | 98.718% | 1.282% | 0.000% | 0.000% |  |  |  |
|  |  | BrS | 90 | -9915.62 | 9.567 | BG: 0.00360 | BG: 1 | BG: 0.00360 | BG: 1 | -1.6E-05 | 0.999 |  |
|  |  |  |  |  |  | FG: 0.00360 | FG: 1 | FG: 1 | FG: 1 |  |  |  |
|  |  |  |  |  |  | 98.718% | 1.282% | 0.000% | 0.000% |  |  |  |
