# Supplementary material for: Host hybridization enabled the emergence of a reassorted hantavirus lineage
Source: PLoS Pathog. 2026 Jul 28;22(7):e1014458. doi: 10.1371/journal.ppat.1014458 (PMC13411931; doi:10.1371/journal.ppat.1014458)
Supplement: S9 Table — All segments from the TULV-CEN.N and TULV-EST.N genomes of the Saxony transect were analysed individually. Additionally, we also analysed the M-segment of the cluster of TULV-CEC & CEE-1 combined and the cluster of TULV-CEE-2 and parental TULV-EST.N combined for evidence of positive or purifying selection within the clade. Positively selected codons are indicated with the posterior probability P (> 0.85) for FUBAR or a p-value (< 0.1) for MEME. (DOCX) [file ppat.1014458.s015.docx]

**S9** **Table: Results of the analysis for signatures of selection with the MEME and FUBAR methods in HYPHY for the TULV S-, M- & L-segments.** All segments from the TULV-CEN.N and TULV-EST.N genomes of the Saxony transect were analysed individually. Additionally, we also analysed the M-segment of the cluster of TULV-CEC & CEE-1 combined and the cluster of TULV-CEE-2 and parental TULV-EST.N combined for evidence of positive or purifying selection within the clade. Positively selected codons are indicated with the posterior probability *P* (> 0.85) for FUBAR or a *p*-value (< 0.1) for MEME.

| All TULV-CEN.N & TULV-EST.N |  |  |
| --- | --- | --- |
| Segment | FUBAR (*P* >= 0.85) | MEME (*p* =< 0.1) |
| S | - | 132 (0.1) |
| M | 18 (0.976) | 1121 (0.05) |
|  | 15 (0.858) | 3 (0.06) |
|  |  | 10 (0.06) |
|  |  | 18 (0.1) |
| L | 1355 (0.963) | 241 (0.01) |
|  |  | 201 (0.04) |
|  |  | 775 (0.04) |
|  |  | 1493 (0.04) |
|  |  | 315 (0.05) |
|  |  | 408 (0.06) |
|  |  | 1355 (0.06) |
|  |  | 1939 (0.06) |
|  |  | 393 (0.08) |
|  |  | 754 (0.08) |
|  |  | 353 (0.09) |
|  |  | 109 (0.1) |
|  |  | 392 (0.1) |
|  |  | 426 (0.1) |
|  |  | 1251 (0.1) |
|  |  | 1833 (0.1) |
|  |  |  |
| TULV-EST.N only |  |  |
| Segment | FUBAR (*P* >= 0.9) | MEME (*p* =< 0.1) |
| M | 18 (0.948) | 696 (0.02) |
|  | 15 (0.935) | 888 (0.1) |
