# Supplementary material for: Host hybridization enabled the emergence of a reassorted hantavirus lineage
Source: PLoS Pathog. 2026 Jul 28;22(7):e1014458. doi: 10.1371/journal.ppat.1014458 (PMC13411931; doi:10.1371/journal.ppat.1014458)
Supplement: S10 Table — Distances were averaged across both the N-terminal ectodomain only and the full length of the protein (in brackets). Only four out seven protein models with identical subunit rotational symmetry were included in the evaluation of structural alignments. (DOCX) [file ppat.1014458.s016.docx]

**S10** **Table: Root mean square deviations RMSDs (Å) of the N-terminal ectodomains of the TULV glycoprotein compared to complete homology models.** Distances were averaged across both the N-terminal ectodomain only and the full length of the protein (in brackets). Only four out seven protein models with identical subunit rotational symmetry were included in the evaluation of structural alignments.

|  | TULV-CEN.N-2 | TULV-CEC-1 | TULV-CEC-2 |
| --- | --- | --- | --- |
| TULV-EST.S | 6.65 (1.38) Å | 7.31 (1.42) Å | 7.34 (1.42) Å |
| TULV-CEN.N-2 |  | 7.55 (1.02) Å | 10.26 (1.21) Å |
| TULV-CEC-1 |  |  | 10.5 (1.32) Å |
